# Protective immune barrier against hepatitis B is needed in individuals born before infant HBV vaccination program in China

**DOI:** 10.1038/srep18334

**Published:** 2015-12-14

**Authors:** Shigui Yang, Chengbo Yu, Ping Chen, Min Deng, Qing Cao, Yiping Li, Jingjing Ren, Kaijin Xu, Jun Yao, Tiansheng Xie, Chencheng Wang, Yuanxia Cui, Cheng Ding, Guo Tian, Bing Wang, Xiaoyan Zhang, Bing Ruan, Lanjuan Li

**Affiliations:** 1 State Key Laboratory for Diagnosis and Treatment of Infectious Diseases, Collaborative Innovation Center for Diagnosis and Treatment of Infectious Diseases, The First Affiliated Hospital, College of Medicine, Zhejiang University, Hangzhou 310003, China; 2 Zhejiang Institute of Medical-care Information Technology, Hangzhou 311112, China; 3 Zhejiang Provincial Center for Disease Control and Prevention, Hangzhou 310051, China

## Abstract

The hepatitis B prevalence rate in adults is still at a high to intermediate level in China. Our purpose was to explore the incidence rate and protective immune barrier against hepatitis B in adults in China. A sample of 317961 participants was multi-screened for hepatitis B surface antigens (HBsAg) in a large-scale cohort of the National Hepatitis B Demonstration Project. A total of 5401 persons were newly-infected, representing an incidence rate of 0.81 (95% CI: 0.77–0.85) per 100 person-years after adjusted by gender and age. History of acquired immune deficiency syndrome, birth prior to 1992, coastal residence, family history of HBV, and migrant worker status were significantly associated with higher incidence, while HBV vaccination and greater exercise with lower incidence. The hepatitis B surface antibody (HBsAb) positive rate was negatively correlated with the incidence rate of hepatitis B (r = −0.826). Linear fitting yielded an incidence rate of 1.23 plus 0.02 multiplied by HBsAb positive rate. The study firstly identified the HBsAg incidence rate, which was reduced to 0.1 per 100 person-years after vaccination coverage of about 64%. The protective immune barrier against hepatitis B needs to be established in individuals born prior to the advent of infant HBV vaccination.

A third of the world’s population is infected with HBV^1^,^2^ and is a major public health threat in the Asia-Pacific region^3^,^4^. HBV vaccination is the most effective prevention strategy recommended by the World Health Organization^3^,^5^,^6^. Many countries had introduced hepatitis B vaccine into their national schedules since the late 1980s^7^,^8^. Among the Vaccine European New Integrated Collaboration Effort (VENICE) network in the European Union, 10 countries have hepatitis B mandatory vaccination^9^. Southeast Asia experienced a significant reduction in HBsAg prevalence between 1990 and 2005, particularly in the age group of 0 to 14 years, with prevalence levels reduced to 1.2%–1.4% in 2005^10^. The Chinese government has conducted hepatitis B vaccination in infants for more than 20 years. As a result, HBsAg prevalence rate has declined significantly, especially in the population born after the implementation of the measures in 1992. Among individuals aged below 20 years, HBsAg prevalence rate decreased from over 10% in 1992^11^ to under 2% in 2006^12^.

However, more than 350 million chronic hepatitis B carriers exist, 75% of whom reside in the Asia-Pacific region^3^. The adult population of Southeast Asia has a higher-to-intermediate level of HBsAg prevalence ranging from 5% to over 6%^10^. In China, the prevalence of HBsAg was 8% to 12% among individuals aged 20 years and older^12^. According to the China Statistical Yearbook 2014^13^, over 1.15 billion were adults aged more than 20 years of the total 1.36 billion adult population in the country, with the number of HBsAg carriers estimated to reach 136 million eventually. Therefore, prevention and control of hepatitis B in adults is a huge challenge. A possible reason for the increased prevalence rate in adults might be attributed to the inadequate protective immune barrier against hepatitis B in the population born before the advent of infant HBV vaccination program. A second factor might be associated with the resistance to adult vaccination measures. Incidence rate might be a better indicator of the effectiveness of hepatitis B vaccination in this population. In contrast to the plethora of data on prevalence, studies exploring the incidence rate are limited^4^,^14^. To assess and reduce the incidence rate of hepatitis B in China, the National Medium and Long-term Science and Technology Development Plan (2006–2020)^15^ emphasized the implementation of a special hepatitis B project. In the project, 12 demonstration zones in Zhejiang, China were selected for a comprehensive hepatitis B prevention and control program. Though other published studies have reported factors that are associated with prevalence rates in China^16^, no evidence exists to examine factors that are associated with newly incident infection risk. This work is the first to observe whether the same factors are present in newly incident cases. Understanding risk factors may help to tailor vaccination programs at those with greater risk profiles and we therefore undertook this work to fill this important gap and identify which characteristics are associated with higher risk of becoming infected.

Based on data collected from the project in the past 6 years, we have analyzed the incidence rate of hepatitis B virus and its risk factors for the first time. The objective of this study was to evaluate the role of immune barrier against hepatitis B in adults, and its implications for accelerated control of hepatitis B infection.

## Results

Table 1 presents the results of prevalence rate analysis. We screened 317961 persons (128344 males and 189617 females) for HBsAg more than twice. Subjects’ age ranged from 0 to 81 years. Of the 317961 individuals screened, 21425 were HBsAg positive, yielding a prevalence rate of 6.74% (95% CI: 6.65%–6.83%). Of the 128344 males tested, 9881 were HBsAg-positive, with a prevalence rate of 7.70% (95% CI: 7.55%–7.85%). Of the 189617 females, 11544 were HBsAg-positive, with a prevalence rate of 6.09% (95% CI: 5.98%–6.20%). The HBsAg prevalence rate in the male group was significantly higher than in the female group (P < 0.01). HBsAg prevalence rate varied significantly among different age groups (P < 0.01). HBsAg prevalence rate of the group aged 0–4 years was the lowest, with a rate of 0.26% (95% CI: 0–0.77%), and the highest in the age group of 35–39 years, with a rate of 9.33% (95% CI: 8.93%–9.73%).The HBsAg prevalence rate in the whole population was standardized by gender and age, with an adjusted rate of 6.22% (95% CI: 6.13%–6.30%). Among 21425 persons with HBsAg positive, there were 57 acute hepatitis B (a prevalence rate of 179.27 per million), 852 chronic hepatitis B (a prevalence rate of 2679.57 per million), 26 severe hepatitis B (a prevalence rate of 81.77 per million), 31 liver cirrhosis resulting from chronic hepatitis B (a prevalence rate of 97.50 per million), and 6 hepatocarcinoma resulting from chronic hepatitis B (a prevalence rate of 18.87 per million).

The results of incidence rate are presented in Table 2. Of the 317961 subjects undergoing HBsAg screening twice or more, the average interval of follow-up was 1.97 years and the total person-years of follow-up was 626383.17. A total of 5401 people were found newly infected, with an incidence rate of 0.86 (95% CI: 0.83–0.89) per 100 person-years. Of the 128344 screened males, the average interval of follow-up was 1.98 years and the total person-years of follow-up was 254121.12, among which 2483 people were found newly infected, with an incidence rate of 0.98 (95% CI: 0.92–1.03) per 100 person-years. Of the 189617 screened females, the average interval of follow-up was 1.97 years and the total person-years of follow-up was 373545.49, of which 2918 people were newly infected, with an incidence rate of 0.78 (95% CI: 0.74–0.82) per 100 person-years. The incidence rate in the male group was significantly higher than that of the female group (P < 0.05). The incidence rates were also significantly different among the different age groups (P < 0.05). In the group aged 0–4 years, the incidence rate was the lowest, with a rate of 0; and the group aged 30–34 years was the highest, with a rate of 1.19 (95% CI: 0.98–1.40) per 100 person-years. The incidence rate in the whole population standardized by gender and age was 0.81 (95% CI: 0.77–0.85) per 100 person-years.

**Figure 1:**
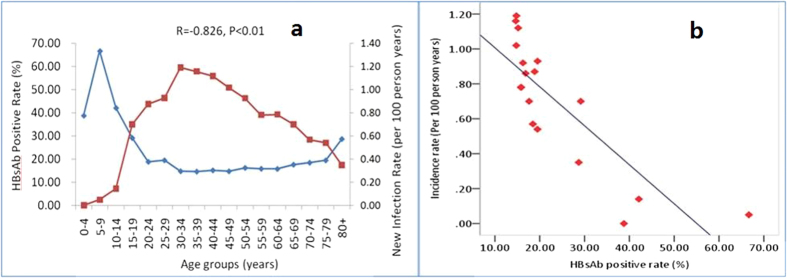
The incidence and hazard ratio of birth prior to 1992. (**a**) The incidence rates of population born before vs. after 1992. The incidence in individuals born before 1992 was four-fold higher than that of those born after 1992. (**b**) The hazard ratio of populations born before and after 1992. The blue line indicated population born after 1992, and the green one population born before 1992.

The analysis of risk factors is presented in Table 3. A history of AIDS and birth prior to 1992, i.e., before the infant HBV vaccination program was implemented, were significantly associated with higher incidence, with RRs (Relative Risk) of 6.09 (95% CI: 1.42–26.17) and 4.06 (95% CI: 2.87–5.76), respectively. After adjustment for gender, age and follow-up interval, the RRs were 5.77 (95% CI: 1.34–24.90, P < 0.01) and 4.35 (95% CI: 3.07–6.16, P < 0.01), respectively. In addition, coastal residence, a family history of hepatitis B, and migrant worker status were also significantly associated with higher incidence, with adjusted RRs of 2.52 (95% CI: 2.39–2.67), 1.73 (95% CI: 1.47–2.02) and 1.75 (95% CI: 1.52–2.01), respectively. HBV vaccination and greater exercise were significantly associated with lower incidence, with adjusted RRs of 0.85 (95% CI: 0.79–0.90) and 0.93 (95% CI: 0.90–0.96), respectively. A significant difference in the incidence of hepatitis B was found between those born before and after 1992 when infant HBV vaccination program was implemented. The incidence rate of hepatitis B was 0.88 (95% CI: 0.85–0.91) per 100 person-years among individuals born before 1992, and 0.21 (95% CI: 0.10–0.31) per 100 person-years among those born after 1992 (Fig. 1a and Supplementary Table 1). The hazard ratio indicated that the incidence was significantly higher among those born before 1992, especially after 60 months, with an adjusted hazard ratio of 8.04 (95% CI: 5.60–11.53) per 100 person-years by gender and age (Fig. 1b and Supplementary Table 1).

**Figure 2:**
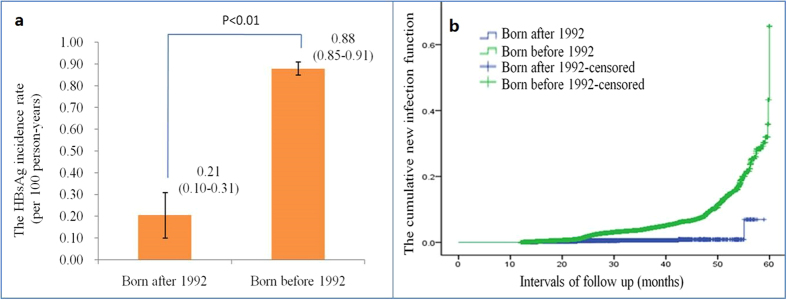
The HBsAg incidence rate of population born in different eras varied with gender and residence. Panel (**a**) The HBsAg incidence rate before and after hepatitis B vaccination of infants since 1992 (Gender); Panel (**b**) The HBsAg incidence rate before and after hepatitis B vaccination of infants since 1992 (Area); Panel (**c**) The relationship between birth era and HBsAg incidence rate; Panel (**d**) The relationship between birth era and susceptibility to hepatitis B infection.

A comparative analysis of rates of HBsAg prevalence and hepatitis B incidence before and after 1992 indicated a strong impact of HBV vaccination program (Fig. 2 and Supplementary Figure 1). HBsAg prevalence rate was reduced dramatically in both male and female population (from 7.9% to 1.8%, and 6.2% to 1.3%, respectively). A similar rate of decline was found upon analysis of the two rates by residence. For both inland and coastal population, HBsAg prevalence rate was reduced significantly after 1992 (from 5.7% to 2.6% and 8.9% to 1.5%, respectively). Remarkably, the HBsAg prevalence rate for coastal population was reduced much faster than that of inland so the rate was much lower (1.5% vs. 2.6%). This reversal was a clear evidence of the effectiveness of HBV vaccination program in the coastal population. This finding is further corroborated by the fact that people born after 1990 had a much lower HBsAg prevalence rate (2.4% after 1990, and less than 1% after 2000) and lower susceptibility rate (carrying hepatitis B antibody). While the hepatitis B incidence rate shared a similar pattern of significant decline after HBV vaccination program implementation in 1992, the reduced incidence rate of male population after 1992, lower than that of the female population (0.27% vs. 0.30%) was remarkable.

**Figure 3:**
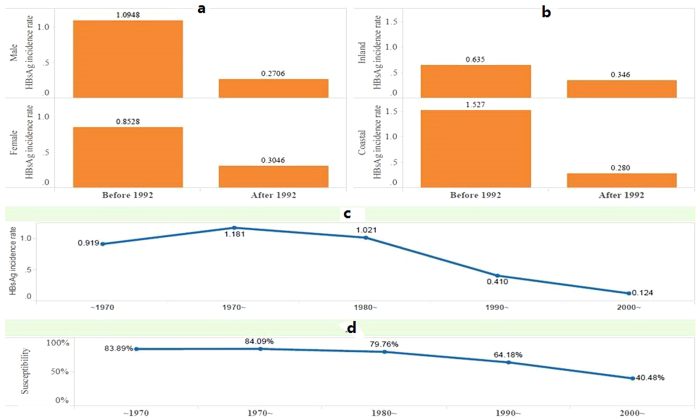
The relationship between HBsAb positive rate and hepatitis B incidence rate. (**a**) Age distribution of HBsAb-positive and hepatitis B incidence rates; (**b**) Scatter diagram and linear fitting for HBsAb-positive rate and the hepatitis B incidence rate, and the incidence rate (ranging from zero to 1.19, per 100 person-years) = 1.23–0.02×the HBsAb positive rate (%).

The HBsAb prevalence rate varied significantly among different age groups (P < 0.01). In particular, the HBsAb positive rate was the highest at 66.67% in the age group of 5–9 years, and the lowest in the group aged 35–39 years, with a rate of 14.64% (Fig. 3a). A strong negative correlation existed between the HBsAb positive rate and the hepatitis B incidence rate, with a correlation coefficient of −0.826 (P < 0.01). A linear fitting for HBsAb positive rate and hepatitis B incidence rate, yielded a hepatitis B incidence rate of 1.23 plus 0.02 times of the HBsAb positive rate (Fig. 3b).

## Discussion

Hepatitis B virus (HBV) infection remains a severe public health problem worldwide^17^,^18^, but its prevalence differs greatly, geographically^10^,^19^,^20^,^21^,^22^. In China, the HBsAg carrier rate was 8.75% in 1979^23^, 9.75% in 1992^24^, and 7.18% in 2006^12^, which is significantly higher than that of the Western countries^8^,^25^. The Chinese government has implemented a plan to control and reduce HBsAg carrier rate, including increased number of intervention projects and funding^26^. Zhejiang province reported the average prevalence level of hepatitis B among Chinese provinces, with an HBsAg carrier rate varying from 5% in inland to 13% in coastal areas^27^. A comprehensive demonstration zone for prevention and control of hepatitis B was established in Zhejiang province since 2009. The results shown in this study indicate that a standardized HBsAg prevalence rate was 6.22% (95% CI: 6.13%–6.30%). The HBsAg prevalence rate in the male group was significantly higher than in the female group. In this study, we had identified the prevalence rates of acute hepatitis B (179.27 per million), chronic hepatitis B (2679.57 per million), severe hepatitis B (81.77 per million), liver cirrhosis resulting from chronic hepatitis B (97.50 per million), and hepatocarcinoma resulting from chronic hepatitis B (18.87 per million). The related diseases resulting from HBV infection remains a severe challenge in China, thus greater importance should be attached to prevention and control of hepatitis B, especially construction of protective immune barrier against hepatitis B in susceptible individuals.

As a time-effective index, the incidence rate directly reflects the threat and effectiveness of preventing and controlling hepatitis B. The prevalence of hepatitis B has been reported to be high in China, but the current incidence level of hepatitis B warrants urgent attention^26^,^28^. In the comprehensive demonstration of zones for prevention and control of hepatitis B^28^, a dynamic epidemiological survey and follow-up for hepatitis B have been carried out more than twice. The results indicated that the standardized incidence rate was 0.81 (95% CI: 0.77–0.85) per 100 person-years. It was 0.98 (95% CI: 0.92–1.03) per 100 person-years in males, higher than that of females, with an incidence rate of 0.78 (95% CI: 0.74–0.82) per 100 person-years.

HBV vaccination is the mainstay of HBV prevention and is highly cost-effective for preventing chronic HBsAg infection in both resource-rich and -limited settings^29^,^30^. In 1992, the Global Advisory Group for the Expanded Program on Immunization of World Health Organization (WHO) recommended^31^ universal HBV vaccination starting from 1995 in countries with high HBV endemicity. The Chinese government was the first nation to institute hepatitis B vaccination measures in infants, in 1992^12^. The rates of prevalence^27^ and incidence decreased in the lower age groups due to infant HBV vaccination. In 2006, the HBsAg carrier rate met the WHO criteria (less than 1%) in the population aged below 5 years after infant vaccination program^8^. The current study suggested that both the incidence and prevalence rates were very low, close to zero and 0.26% (95% CI: 0–0.77%) in the age groups of 0 to 4 years, respectively. The reductions were seen in groups aged about 20 years, with the incidence rate of hepatitis B of 0.21 (95% CI: 0.10–0.31) per 100 person-years in population born after 1992, indicating that those age groups developed a strong protective barrier of immunity. Further, the decreased incidence rate in the lower age group was attributed to a lack of similar exposure to HBV compared with the older age groups^32^. However, compared with the HBV vaccination program, the role of HBV exposure was not significant, as it was indicated in a serological investigation before the era of the national HBV vaccination program in 1992 when the HBsAg carrier rate was unexpectedly high at a rate of over 9% in lower age group^27^.

However, the HBV vaccination strategy among adults is not yet active in China^33^, as well as in other countries^6^,^7^,^8^,^34^. The coverage rate of HBV vaccination is very low in paying adults in China^33^, and hepatitis B still remains a severe public health challenge in adults. Our results indicated that the incidence rate was nearly 1.19 (95% CI: 0.98–1.40) per 100 person-years, and the prevalence rate reached 9.33% (95% CI: 8.93%–9.73%) in the age group of 35–39. We found that those born before 1992 showed the strongest risk of incidence of hepatitis B, with adjusted Relative Risk (RR) of 4.35 (95% CI: 3.07–6.16). The incidence rate of hepatitis B was 0.88 (95% CI: 0.85–0.91) per 100 person-years in those born before 1992, which was more than four times the rates after 1992. Compared with the population born after 1992, the hazard ratio of hepatitis B incidence in the population born before 1992 was 8.04 (95% CI: 5.60–11.53) per 100 person-years. Therefore, individuals born before 1992 were highly vulnerable to hepatitis B virus, especially among those aged 35–40 years. The rates of HBsAg prevalence and incidences were most dramatically reduced among coastal and male populations. Assuming over 1.15 billion adults were aged greater than 20 years among the 1.36 billion adults in China^13^, the number of new infections is expected to climb to 10.12 million per year. Therefore, it is imperative to implement the HBV vaccination program and construct a protective immune barrier, which is the only way to comprehensively control the hepatitis B epidemic in this population. Setting a 0.1% incidence rate as the threshold for controlling hepatitis B in a population, a linear model of this study suggests that the target can be accomplished only when HBsAb positive rate in the adult population exceeded 56.5%. Assuming an effective rate of 88% after a three-dose HBV vaccination^35^, the 56.5% HBsAb positive rate requires a vaccine coverage rate of 64% of the adult population.

Understanding risk factors present in newly infected cases may help us to tailor vaccination programs at those with greater risk profiles. Our study found that a history of AIDS, birth prior to 1992, coastal residence, a family history of HBV, and migrant worker status were significantly associated with higher incidence, while HBV vaccination and greater exercise with lower incidence. A study in South India indicated that cultural practices, such as tattooing, traditional medicine (e.g., blood-letting), rituals (e.g., scarification), and body-piercing, also potentially increased the risk of HBV transmission^36^. The sex distribution are consistent with most published studies where male were higher^37^. Our previous study^27^ reported that a family history of hepatitis B, drinking, smoking, migrant worker status, and/or specific occupations (fishermen, construction workers, and long-distance truck drivers) were important risk factors affecting HBsAg prevalence. A retrospective observational cohort study indicated a higher rate of hepatitis B virus (HBV) co-infection in HIV infection^38^, which confirmed HIV infection as a significant risk factor for HBsAg incidence rate. HBV was transmitted by practices such as unsafe injection or immunodeficiency similar to HIV infection. While high risk and protective factors for HBsAg prevalence were studied by other researchers in the past, factors influencing incidence rate were analysed in this study for the first time. It is important to acknowledge that causality cannot be inferred from observational analyses and significant associations may simply reflect confounding, for example, those with lower exercise levels or a history of AIDS may also have other high-risk behavior that leads to infection; though the higher risk of infection in those born prior to the introduction of the vaccination program seems likely to be a direct result.

In conclusion, the HBsAg incidence rate was analyzed and a set of influencing factors identified in this study for the first time. From a public health perspective, incidence rate is a more important consideration for the design of health strategies and education campaigns for preventing HBV transmission. Based on the study results, the HBV vaccination program of 1992 in China is effective in populations born after 1992 across different age groups. The reduction was most dramatic among coastal and male populations. If 0.1% incidence rate is set as the target for population control of hepatitis B, the study found that this can be attained only when HBsAb-positive rate was over 56.5% in adult population. An effective rate of 88% after a three-dose HBV vaccination^35^, requires HBV vaccination of 64% of the adult population. The large sample size, prospective nature of the study and detailed baseline data permit this to be examined in a comprehensive way and we are confident that these findings may be used for targeting vaccination programs at those with greater risk for infection, thus making optimum use of resources.

## Methods

The technical design of this large-scale cohort study of the national hepatitis B demonstration project was based on standard operation procedures (SOP)^28^. In brief, 12 Hepatitis B prevention and control demonstration zones were established in Zhejiang, China. The activities consist of the following: (1) multiple and large-scale screenings for hepatitis B (HBsAg) conducted every two years; (2) prevention measures including vaccination and health education of uninfected population, comprehensive normalized treatment and management measures for infected population; (3) a cloud-based data system established to collect and store epidemiological data of infectious diseases along with a standard set of documents^28^, covering personal electronic health, personal physical examination, patients with hepatitis B, and follow-up of patients with hepatitis B; and (4) monitoring the normalization of procedure and data accuracy, independent quality control and verification measures, to ensure the accuracy and consistence of the data^39^.

The cohort follow-up was open, and participants were eligible if they had HBsAg results recorded in the database more than twice from Jan 1, 2009 to Dec 31, 2014. The study was approved by the Ethics Committee of The First Affiliated Hospital at the School of Medicine of Zhejiang University.

**Figure 4:**
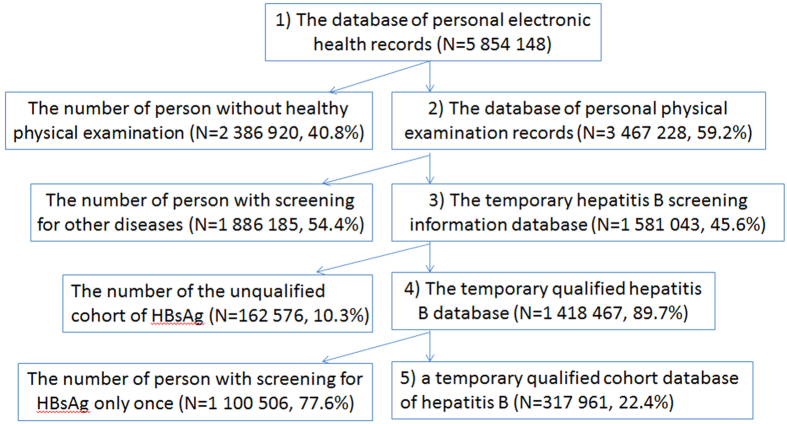
Flow chart outlining data assessment and population selection.

Samples were selected using the following four steps. First, individual citizen IDs were used to merge records from a personal electronic health record database (N = 5854148) and personal physical examination records (N = 3467228) database, to create a preliminary hepatitis B database. Since only the data records containing complete personal baseline data and hepatitis B screening information were selected, the database size was reduced to 1581043. Second, the selected records were filtered by scrutinizing for unqualified data pertaining to gender, birth date, screening date, and other parameters to yield an improved database (N = 1418 467). Third, in order to determine the new infection incidence, only records with both initial screening and follow-up were selected to obtain the study data set, which further reduced the database size to 317961 records (Fig. 4).

We assessed the new cases of infection based on those negative for HBsAg in the first round of screening and positive in the last round of screening. We also assessed the individual follow-up period (years) based on the time since the date of the first screening to the last round of screening.

To assess hepatitis B incidence rate and the level of immune barrier against hepatitis B, we selected two cohorts. The adult cohort consisted of participants born before 1992, none of them covered by infant HBV vaccination program. The cohort of children and teenagers consisted of participants born after 1992, mostly covered by infant HBV vaccination program. To assess the factors influencing hepatitis B incidence rate, 31 variables related to hepatitis B were finally selected and these related to socio-economics, lifestyle and medical history (Supplementary Table 2).

To assure the sample representativeness in the study database (N = 317961), a comparative analysis of social demography and health information was conducted to evaluate the differences between population and the study sample. The variables used include age, gender, residence, mobility, family history, past medical history, personal history, and the history of vaccination.

All the data were stored in an Oracle database and analyzed using SAS statistical analysis software (version 9.4). The prevalence rate of hepatitis B was defined as the number of HBsAg-positive people divided by the total number of people screened for HBsAg at a given time. The incidence rate of hepatitis B (per 100 person-years) was measured in terms of the number of HBsAg-negative people in the first round of screening but testing positive in the last round of screening divided by the product of the number of people screened for HBsAg twice and the follow-up interval (years). The total population rates were standardized by the age and gender composition based on the normal population^40^. The factors influencing hepatitis B incidence rate were analyzed using multivariate logistic regression. The accumulated function of the incidence risk trend was annualized using Kaplan-Meier method. The incidence risk (hazard ratio, HR) of hepatitis B among different generations was analyzed using Cox model. The results were adjusted by age and gender.

## Additional Information

**How to cite this article**: Yang, S. *et al*. Protective immune barrier against hepatitis B is needed in individuals born before infant HBV vaccination program in China. *Sci. Rep*. **5**, 18334; doi: 10.1038/srep18334 (2015).

## Supplementary Material

Supplementary Information

## Figures and Tables

**Table 1:** HBsAg prevalence rate in different genders and age groups.

Groups	Sample Size	No. of HBsAg-Positive individuals	HBsAg Prevalence Rate (%)	95% CI for Prevalence Rate (%)
Males	128344	9881	7.70	7.55–7.85
Females	189617	11544	6.09	5.98–6.20
0–4	384	1	0.26	0–0.77
5–9	1778	6	0.34	0.07–0.61
10–14	3700	49	1.32	0.95–1.70
15–19	2347	100	4.26	3.43–5.10
20–24	3547	227	6.40	5.57–7.23
25–29	5747	424	7.38	6.68–8.08
30–34	10512	885	8.42	7.86–8.97
35–39	22127	2064	9.33	8.93–9.73
40–44	34751	2989	8.60	8.29–8.91
45–49	39821	3246	8.15	7.87–8.43
50–54	41716	3346	8.02	7.75–8.29
55–59	46647	3014	6.46	6.23–6.69
60–64	41562	2330	5.61	5.38–5.83
65–69	25089	1236	4.93	4.65–5.20
70–74	21952	875	3.99	3.72–4.25
75–79	15087	600	3.98	3.66–4.30
80+	1194	33	2.76	1.82–3.71
All	317961	21425	6.74	6.65–6.83
Standardized*	–	–	6.22	6.13–6.30

^*^Adjusted by gender and age of the standardized population.

**Table 2:** HBV (HBsAg) incidence rate in different genders and age groups.

Groups	Sample size	No. of new infections	Average interval of follow–up (years)	Total person-years of follow-up	Incidence rate*	95% CI for Incidence rate (%)*
Males	128344	2483	1.98	254121.12	0.98	0.92–1.03
Females	189617	2918	1.97	373545.49	0.78	0.74–0.82
0–4	384	0	2.78	1067.52	0.00	0.00–0
5–9	1778	2	2.31	4107.18	0.05	0–0.15
10–14	3700	11	2.06	7622.00	0.14	0.02–0.27
15–19	2347	32	1.95	4576.65	0.70	0.36–1.04
20–24	3547	63	2.03	7200.41	0.87	0.57–1.18
25–29	5747	109	2.05	11781.35	0.93	0.68–1.18
30–34	10512	255	2.04	21444.48	1.19	0.98–1.40
35–39	22127	512	2.00	44254.00	1.16	1.01–1.30
40–44	34751	766	1.97	68459.47	1.12	1.01–1.23
45–49	39821	791	1.96	78049.16	1.02	0.92–1.11
50–54	41716	758	1.97	82180.52	0.92	0.83–1.02
55–59	46647	718	1.97	91894.59	0.78	0.70–0.86
60–64	41562	632	1.94	80630.28	0.78	0.70–0.87
65–69	25089	341	1.95	48923.55	0.70	0.60–0.80
70–74	21952	246	1.98	43464.96	0.57	0.47–0.67
75–79	15087	158	1.94	29268.78	0.54	0.42–0.66
80+	1194	7	1.69	2017.86	0.35	0.01–0.68
All	317961	5401	1.97	626383.17	0.86	0.83–0.89
Standardized#					0.81	0.77–0.85

^*^Per 100 person-years.

^#^Adjusted by gender and age of the standardized population.

**Table 3:** Factors affecting HBV (HBsAg) incidence rate.

Variables	OR	OR 95% CI	P	OR(adj)*	95% CI for OR(adj)	P
1. Coastal residence	2.42	2.29–2.56	<0.001	2.52	2.39–2.67	<0.001
Inland residence	1.00			1.00		
2. Birth before 1992	4.06	2.87–5.76	<0.001	4.35	3.07–6.16	<0.001
Birth after 1992	1.00			1.00		
3. HB Vaccination	0.79	0.74–0.85	<0.001	0.85	0.79–0.90	<0.001
No HB Vaccination	1.00			1.00		
4. Family history of HB	1.54	1.32–1.79	<0.001	1.73	1.47–2.02	<0.001
No family history of HB	1.00			1.00		
5. History of AIDS	6.09	1.42–26.17	0.015	5.77	1.34–24.90	0.019
No history of AIDS	1.00			1.00		
6. Exercises (Grade)	0.94	0.91–0.97	<0.001	0.93	0.90–0.96	<0.001
No exercise	1.00			1.00		
7. Migrant worker status	1.95	1.70–2.24	<0.001	1.75	1.52–2.01	<0.001
Local resident status	1.00			1.00		

^*^Adjusted by gender, age and time of follow-up.

## References

[b1] EASL clinical practice guidelines: Management of chronic hepatitis B virus infection. J Hepatol 57, 167-85 (2012). 22436845 10.1016/j.jhep.2012.02.010

[b2] Hepatitis B vaccines: WHO position paper—recommendations. Vaccine 28, 589-90 (2010). 19896455 10.1016/j.vaccine.2009.10.110

[b3] Liaw Y. F. & Chu C. M. Hepatitis B virus infection. Lancet 373, 582–92 (2009). 19217993 10.1016/S0140-6736(09)60207-5

[b4] Howell J., Van Gemert C., Lemoine M., Thursz M. & Hellard M. An overview of hepatitis B prevalence, prevention, and management in the Pacific Islands and Territories. J Gastroenterol Hepatol 29, 1854–66 (2014). 25131570 10.1111/jgh.12684

[b5] Beasley R. P. Development of hepatitis B vaccine. JAMA 302, 322–4 (2009). 19602694 10.1001/jama.2009.1024

[b6] Rani M., Yang B. & Nesbit R. Hepatitis B control by 2012 in the WHO Western Pacific Region: rationale and implications. Bull World Health Organ 87, 707–13 (2009). 19784451 10.2471/BLT.08.059220 PMC2739917

[b7] Progress towards meeting the 2012 hepatitis B control milestone: WHO Western Pacific Region, 2011. *Wkly Epidemiol Rec* **86**, 180-8 (2011). 21608201

[b8] Hennessey K., Mendoza-Aldana J., Bayutas B., Lorenzo-Mariano K. M. & Diorditsa S. Hepatitis B control in the World Health Organization’s Western Pacific Region: targets, strategies, status. Vaccine 31 Suppl 9, J85–92 (2013). 24331026 10.1016/j.vaccine.2012.10.082

[b9] Haverkate M. . Mandatory and recommended vaccination in the EU, Iceland and Norway: results of the VENICE 2010 survey on the ways of implementing national vaccination programmes. Euro Surveill 17 (2012). 10.2807/ese.17.22.20183-en 22687916

[b10] Ott J. J., Stevens G. A., Groeger J. & Wiersma S. T. Global epidemiology of hepatitis B virus infection: new estimates of age-specific HBsAg seroprevalence and endemicity. Vaccine 30, 2212–9 (2012). 22273662 10.1016/j.vaccine.2011.12.116

[b11] Chen Y. S., Wang X. X., Shang P. H. & Liang X. F. The study of tendency of hepatitis B virus surface antigen in Chinese population. Chin J Exp Clin InfectDis (ElectronicVersion) 1, 5 (2007).

[b12] Liang X. . Epidemiological serosurvey of hepatitis B in China–declining HBV prevalence due to hepatitis B vaccination. Vaccine 27, 6550–7 (2009). 19729084 10.1016/j.vaccine.2009.08.048

[b13] The basic situation of the population. (National Bureau of Statistics of China).

[b14] Chu C. M. & Liaw Y. F. HBsAg seroclearance in asymptomatic carriers of high endemic areas: appreciably high rates during a long-term follow-up. Hepatology 45, 1187–92 (2007). 17465003 10.1002/hep.21612

[b15] Ministry of Science and Technology of the People’s Republic of China, National medium and long-term science and technology development plan. (2006) Available at: http://www.most.gov.cn/mostinfo/xinxifenlei/gjkjgh/200811/t20081129_65774.htm. (Accessed:11/ 03/ 2015).

[b16] Li X. . Hepatitis B virus infections and risk factors among the general population in Anhui Province, China: an epidemiological study. BMC Public Health 12, 272 (2012). 22475135 10.1186/1471-2458-12-272 PMC3355038

[b17] Liu J. & Fan D. Hepatitis B in China. Lancet 369, 1582–3 (2007). 17499584 10.1016/S0140-6736(07)60723-5

[b18] Torpy J. M., Burke A. E. & Golub R. M. JAMA patient page. Hepatitis B. JAMA 305, 1500 (2011). 21486984 10.1001/jama.305.14.1500

[b19] Lee W.M. Hepatitis B virus infection. N Engl J Med 337, 1733–45 (1997). 9392700 10.1056/NEJM199712113372406

[b20] Edmunds W. J. . Epidemiological patterns of hepatitis B virus (HBV) in highly endemic areas. Epidemiol Infect 117, 313–25 (1996). 8870629 10.1017/s0950268800001497 PMC2271713

[b21] Caley M., Fowler T., Greatrex S. & Wood A. Differences in hepatitis B infection rate between ethnic groups in antenatal women in Birmingham, United Kingdom, May 2004 to December 2008. Euro Surveill 17 (2012). 22856511

[b22] Duffell E. F. & van de Laar M. J. Survey of surveillance systems and select prevention activities for hepatitis B and C, European Union/European Economic Area, 2009. Euro Surveill 20, 17–24 (2015). 25860392 10.2807/1560-7917.es2015.20.13.21080

[b23] Chen Y. S., Wang X. X. & Shang P. H. The study of tendency of hepatitis B virus surface antigen in Chinese population. Chin J Exp Clin Infect Dis 1, 5 (2007).

[b24] Xia G. L. L., C.B. Cao H. L. . Prevalence of hepatitis B and C virus infections in the conventional Chinese population: results from a nationwide cross-sectional seroepidemiologic study of hepatitis A, B, C, D and E virus infections in China, 1992. Int Hepatol Commun 5, 12 (1996).

[b25] Dienstag J. L. Hepatitis B virus infection. N Engl J Med 359, 1486–500 (2008). 18832247 10.1056/NEJMra0801644

[b26] Application guide for science and technology project of a major infectious disease prevention and control (The Ministry of Health of China, 2008).

[b27] Yang S. G. . Effectiveness of HBV vaccination in infants and prediction of HBV prevalence trend under new vaccination plan: findings of a large-scale investigation. PLoS One 7, e47808 (2012). 23094094 10.1371/journal.pone.0047808 PMC3477110

[b28] Li L. (ed.) Standard operation procedure for major projects of national science and technology of infectious disease: the community comprehensive prevention and control of major infectious diseases, 89 (Science Press, Beijing, 2012).

[b29] Aggarwal R., Ghoshal U. C. & Naik S. R. Assessment of cost-effectiveness of universal hepatitis B immunization in a low-income country with intermediate endemicity using a Markov model. J Hepatol 38, 215–22 (2003). 12547411 10.1016/s0168-8278(02)00382-3

[b30] Ni Y. H. . Minimization of hepatitis B infection by a 25-year universal vaccination program. J Hepatol 57, 730–5 (2012). 22668640 10.1016/j.jhep.2012.05.021

[b31] Marion S. A., Tomm Pastore M., Pi D. W. & Mathias R. G. Long-term follow-up of hepatitis B vaccine in infants of carrier mothers. Am J Epidemiol 140, 734–46 (1994). 7942775 10.1093/oxfordjournals.aje.a117321

[b32] Mast E. E. . A comprehensive immunization strategy to eliminate transmission of hepatitis B virus infection in the United States: recommendations of the Advisory Committee on Immunization Practices (ACIP) part 1: immunization of infants, children, and adolescents. MMWR Recomm Rep 54, 1–31 (2005). 16371945

[b33] Cui F. Q. [Technical guide for adult hepatitis B immunization in China]. Zhonghua Liu Xing Bing Xue Za Zhi 32, 1199–203 (2011). 22336599

[b34] Hennessey K. . Are we there yet? Assessing achievement of vaccine-preventable disease goals in WHO’s Western Pacific Region. Vaccine 32, 4259–66 (2014). 24947995 10.1016/j.vaccine.2014.02.093

[b35] Yao J. . The response of hepatitis B vaccination on seronegative adults with different vaccination schedules. Hum Vaccin Immunother 0 (2015). 10.4161/21645515.2014.985500 PMC4514229 25621975

[b36] Chandra M. . Prevalence, risk factors and genotype distribution of HCV and HBV infection in the tribal population: a community based study in south India. Trop Gastroenterol 24, 193–5 (2003). 15164530

[b37] Hansen N. . Hepatitis B prevalence in Denmark - an estimate based on nationwide registers and a national screening programme, as on 31 December 2007. Euro Surveill 18 (2013). 10.2807/1560-7917.es2013.18.47.20637 24300884

[b38] Zhang F. . HIV, hepatitis B virus, and hepatitis C virus co-infection in patients in the China National Free Antiretroviral Treatment Program, 2010-12: a retrospective observational cohort study. Lancet Infect Dis 14, 1065–72 (2014). 25303841 10.1016/S1473-3099(14)70946-6 PMC6051428

[b39] Application guide for science and technology project of a major infectious disease prevention and control (The Ministry of Health of China, 2013).

[b40] the Zhejiang province population sampling survey data the main bulletin in 2012 (Zhejiang Provincial Bureau of Statistics, Hangzhou, 2012).

