# Supplementary material for: Protective immune barrier against hepatitis B is needed in individuals born before infant HBV vaccination program in China
Source: Sci Rep. 2015 Dec 14;5:18334. doi: 10.1038/srep18334 (PMC4677310; doi:10.1038/srep18334)

Supplementary Materials

Title: Protective immune barrier against hepatitis B is needed in individuals born before infant HBV vaccination program in China

Authors: Shigui Yang[1], Chengbo Yu[1], Ping Chen[1], Min Deng[1], Qing Cao[1], Yiping Li[2], Jingjing Ren[1], Kaijin Xu[1], Jun Yao[3], Tiansheng Xie[1], Chencheng Wang[1], Yuanxia Cui[1], Cheng Ding[1], Guo Tian[1], Bing Wang[1], Xiaoyan Zhang[1], Bing Ruan[1*], Lanjuan Li[1*]

[1] State Key Laboratory for Diagnosis and Treatment of Infectious Diseases, Collaborative Innovation Center for Diagnosis and Treatment of Infectious Diseases, The First Affiliated Hospital, College of Medicine, Zhejiang University, Hangzhou310003, China

[2]Zhejiang Institute of Medical-care Information Technology, Hangzhou 311112, China

[3]Zhejiang Provincial Center for Disease Control and Prevention, Hangzhou 310051, China

Correspondence author: Prof. Lanjuan Li, MD. and Bing Ruan

Email: ljli@zju.edu.cn and hzruanbing@163.com

Supplementary Table 1: Incidence and hazard ratios of factors associated with birth before infant HBV vaccination program implementation in 1992

Item	Birth after 1992	Birth before 1992
Population size	7425	310536
The number of new infections	32	5369
Mean follow-up period (years)	15582.12	610976.53
The total number of follow-up person-years	32	5369
The incidence rate (95% CI)*	0.21 (0.10-0.31)	0.88 (0.85-0.91)
Hazard ratio (HR, 95% CI)	4.96 (3.50-7.01)	
Hazard ratio (HR) adjusted by gender and age	8.04 (5.60-11.53)	

* per 100 person-years

Supplementary Table 2: Selection of variables in the cloud-based data system

Field Name	Field Code	Data Format	Fill Mode	Range
Citizen ID	ID_CARD_NO	VARCHAR2 (100 CHAR)	Required	The standard citizen ID number
Date of Check-up	DATE_CHECKUP	TIMESTAMP(6)	Required	
Gender	GENDER	NUMBER(10)	Required	0: Unknown Gender; 1: Males; 2: Females; 9: Unknown
Date of birth	DATE_ BIRTHDAY	TIMESTAMP(6)	Required	
Nation	NATIONALITY	NUMBER(10)	Option	Reference: the GBT 3304-1991 national digital code
Administrative division code	AREA_CODE	NUMBER(19)	Required	Reference to residential address
Occupation	CAREER	NUMBER(10)	Option	1.Officer; 2.Professional and technical personnel; 3.Worker; 4.Business and service personnel; 5.Farmer; 6.Transport-related workers; 7.Soldier; 8.Other;
Education	EDUCATION_LEVEL	NUMBER(10)	Option	1: Illiterate and semi-literate; 2. Primary school; 3. Junior high school; 4. High school /technical school / college; 5. University degree or above; 6. Unknown
Marital status	MARITAL_STATUS	NUMBER(10)	Option	1. Unmarried; 2. Married; 3. Widowed; 4. Divorced; 5. Unspecified marital status.
Out 3 months or more per year	GO_OUT3MONTHS_PER_YEAR	NUMBER(10)	Required	1: No, 2: Yes, 3: Unknown
Smoking habits	SMOKING_HABIT	NUMBER(10)	Option	1. Never smoked; 2: Quit smoking; 3. Smoking.
Drinking frequency	DRINKING_FREQUENCY	NUMBER(10)	Option	1. Never; 2. Occasionally; 3. More than once a week; 4.Every day.
Exercise frequency	EXERCISE_FREQUENCY	NUMBER(10)	Option	1. Every day; 2. More than once a week; 3. Occasionally; 4. Never.
Hepatitis B vaccination history	HB_VACCINATION	NUMBER(10)	Required	1: No, 2: Yes, 3: Unknown
Blood type	BLOOD_TYPE	NUMBER(10)	Option	1：Type A，2: Type B, 3: Type O, 4: Type AB
Genetic history	HAVING_GENETIC_DISEASE	NUMBER(10)	Option	1: No, 2: Yes, 3: Unknown
History of drug allergy	HISTORY_OF_ALLERGY_O	VARCHAR2 (100 CHAR)	Option	Can be selected: 1. Without; 2. Penicillin; 3. Sulfonamide; 4. Streptomycin; 5. Other. The comma separator was used to report multiple values, for example: 1,2,3,4,5
History of trauma	HISTORY_OF_TRAUMA	NUMBER(10)	Option	1: No, 2: Yes, 3: Unknown
Surgery	HISTORY_OF_SURGERY	NUMBER(10)	Required	1: No, 2: Yes, 3: Unknown

History of blood transfusion	HISTORY_OF_TRANSFUSION	NUMBER(10)	Required	1: No, 2: Yes, 3: Unknown
Past history of AIDS	HISTORY_OF_AIDS	NUMBER(10)	Required	1: No, 2: Yes, 3: Unknown
Past history of hepatitis B	HISTORY_OF_HB	NUMBER(10)	Required	1: No, 2: Yes, 3: Unknown
Past history of TB	HISTORY_OF_TB	NUMBER(10)	Required	1: No, 2: Yes, 3: Unknown
Family history of hepatitis B	FAMILY_HISTORY_OF_HB	NUMBER(10)	Required	1: No, 2: Yes, 3: Unknown
Family history of hepatitis B - Father	FATHER_HISTORY_OF_DISEASES_O	VARCHAR2 (100 CHAR)	Required	Can be selected: 1. Without; 2. Hypertension; 3. Diabetes; 4. Coronary heart disease; 5. Chronic obstructive pulmonary disease; 6. Malignant tumor; 7. Stroke; 8:Severe mental illness; 9:Tuberculosis; 10:Hepatitis B; 11. Congenital malformation; 12. AIDS; 13. Other. The comma separator was used to report multiple values, for example: 1,2,3,4, and 5.
Family history of hepatitis B - mother	MOTHER_HISTORY_OF_DISEASES_O	VARCHAR2 (100 CHAR)	Required	Can be selected: 1. Without; 2. Hypertension; 3. Diabetes; 4. Coronary heart disease; 5. Chronic obstructive pulmonary disease; 6. Malignant tumor; 7. Stroke; 8:Severe mental illness; 9:Tuberculosis; 10:Hepatitis B; 11. Congenital malformation; 12. AIDS; 13. Other. The comma separator was used to report multiple values, for example: 1,2,3,4, and 5.
Family history of hepatitis B-siblings	SIBLINGS_HISTORY_OF_DISEASES_O	VARCHAR2 (100 CHAR)	Required	Can be selected: 1. Without; 2. Hypertension; 3. Diabetes; 4. Coronary heart disease; 5. Chronic obstructive pulmonary disease; 6. Malignant tumor; 7. Stroke; 8:Severe mental illness; 9:Tuberculosis; 10:Hepatitis B; 11. Congenital malformation; 12. AIDS; 13. Other. The comma separator was used to report multiple values, for example: 1,2,3,4, and 5.
Family history - children	CHILDREN_HISTORY_OF_DISEASES_O	VARCHAR2 (100 CHAR)	Required	Can be selected: 1. Without; 2. Hypertension; 3. Diabetes; 4. Coronary heart disease; 5. Chronic obstructive pulmonary disease; 6. Malignant tumor; 7. Stroke; 8:Severe mental illness; 9:Tuberculosis; 10:Hepatitis B; 11. Congenital malformation; 12. AIDS; 13. Other. The comma separator was used to report multiple values, for example: 1,2,3,4, and 5.
Hepatitis B surface antibody	HBSAB	NUMBER(10)	Required	1: Negative; 2: Positive; 3: Unknown.
Hepatitis B surface antigen	HBSAG	NUMBER(10)	Required	1: Negative; 2: Positive; 3: Unknown.
Check whether serum alanine aminotransferase	HAVING_SGPT_TEST	NUMBER(10)	Option	Numerical results of laboratory studies, u/l unit

Supplementary figure 1: The HBsAg prevalence rate in individuals born in different eras varied with gender and residence. Panel a: The HBsAg prevalence rate before and after HB vaccination of infants since 1992 (Gender); Panel b: The HBsAg prevalence rate before and after HB vaccination of infants since 1992 (Area); Panel c: The relationship between birth era and HBsAg prevalence rate; Panel d: The relationship between birth era and susceptibility to hepatitis B.

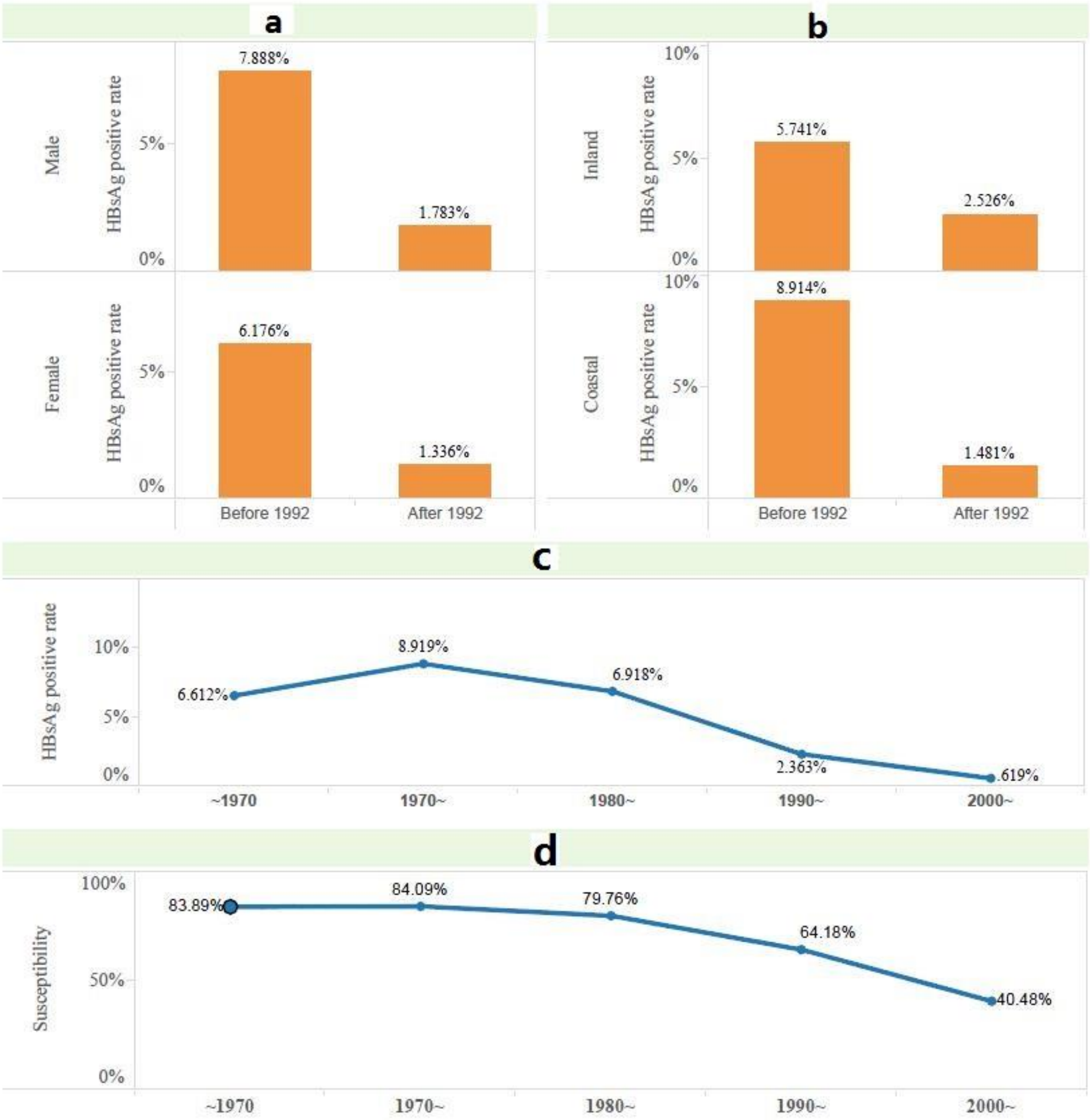

Supplement: Supplementary Information [file srep18334-s1.pdf]
